# The essential role of dual-energy x-ray absorptiometry in the prediction of subclinical cardiovascular disease

**DOI:** 10.3389/fcvm.2024.1377299

**Published:** 2024-08-29

**Authors:** Sisi Yang, Qin Chen, Yang Fan, Cuntai Zhang, Ming Cao

**Affiliations:** Department of Geriatrics, Tongji Hospital, Tongji Medical College, Huazhong University of Science and Technology, Wuhan, China

**Keywords:** cardiovascular risk assessment, subclinical cardiovascular disease, body composition analysis, abdominal aortic calcification, dual-energy x-ray absorptiometry

## Abstract

Subclinical cardiovascular disease (Sub-CVD) is an early stage of cardiovascular disease and is often asymptomatic. Risk factors, including hypertension, diabetes, obesity, and lifestyle, significantly affect Sub-CVD. Progress in imaging technology has facilitated the timely identification of disease phenotypes and risk categorization. The critical function of dual-energy x-ray absorptiometry (DXA) in predicting Sub-CVD was the subject of this research. Initially used to evaluate bone mineral density, DXA has now evolved into an indispensable tool for assessing body composition, which is a pivotal determinant in estimating cardiovascular risk. DXA offers precise measurements of body fat, lean muscle mass, bone density, and abdominal aortic calcification, rendering it an essential tool for Sub-CVD evaluation. This study examined the efficacy of DXA in integrating various risk factors into a comprehensive assessment and how the application of machine learning could enhance the early discovery and control of cardiovascular risks. DXA exhibits distinct advantages and constraints compared to alternative imaging modalities such as ultrasound, computed tomography, magnetic resonance imaging, and positron emission tomography. This review advocates DXA incorporation into cardiovascular health assessments, emphasizing its crucial role in the early identification and management of Sub-CVD.

## 1 Introduction

Subclinical cardiovascular disease (Sub-CVD) is an early stage of cardiovascular disease (CVD) that is frequently asymptomatic. It is commonly observed in patients with sedentary lifestyles, hypertension, diabetes, or obesity, which are all risk factors (1–4). The confluence of health variables and lifestyle behaviors contributes to the development of Sub-CVD. Smoking, physical inactivity, unhealthy diet, and obesity are major contributors to its pathogenesis. High cholesterol levels, specifically low-density lipoprotein cholesterol, hypertension, and poor glucose control, play key roles in developing Sub-CVD, contributing to arterial plaque formation and vascular damage (2, 5). Numerous studies have documented how these behaviors aggravate problems such as arterial stiffness, atherosclerosis, and metabolic abnormalities (1, 2, 6–8). Clinical diseases, including coronary and peripheral artery diseases, can result from Sub-CVDs. At the outset, atherosclerosis develops because of the accumulation of plaques in the arteries. The risk factors include hypertension, obesity, and hypercholesterolemia. Patients may experience heart failure, arrhythmias, valve disorders, and stroke in the later stages (7, 9). CVD represents a major public health concern on a global scale, leading to a great clinical disease burden, high mortality rates, and a substantial financial burden on healthcare systems (10). Timely diagnosis and intervention are crucial for managing and preventing risk factors from developing into clinically apparent cardiovascular conditions.

Recent studies have demonstrated that, besides conventional risk factors, body composition measures such as excessive body fat, sarcopenia, and osteoporosis significantly increase the prevalence of Sub-CVD (11–13). Systemic inflammation and atherogenic dyslipidemia are both effects of visceral fat excess (14, 15). Sarcopenia, characterized by muscle loss and decreased function, is associated with reduced physical activity and an elevated risk of cardiovascular incidents (16, 17). Osteoporosis and Sub-CVD have similar risk factors (12, 18, 19), and osteoporosis may also influence arterial calcium deposition. Furthermore, abdominal aortic calcification (AAC) serves as an indicator of advanced atherosclerosis, which may manifest before coronary artery calcification, be detected in young persons, and reflects an elevated cardiovascular risk (19–24). Mounting data underscores the necessity for further studies and integration of these factors into clinical assessments and therapeutic strategies for cardiovascular health.

Initially employed to determine bone mineral density (BMD), dual-energy x-ray absorptiometry (DXA) (25) has evolved into a valuable tool for evaluating body composition (26). The conventional use of DXA to assess bone density may indirectly indicate cardiovascular risk owing to the correlation between osteoporosis and CVD. Recent studies have highlighted a potential link between bone health and cardiovascular risks. The accuracy of DXA in determining lean muscle mass and body fat assists in detecting excessive visceral fat, a major risk factor for Sub-CVD (27–29). The efficiency of DXA in diagnosing sarcopenia by measuring lean muscle mass highlights its role in cardiovascular risk assessment, given the established link between muscle mass loss and increased cardiovascular risk (16, 30–33). Additionally, DXA can detect AAC, a significant marker for atherosclerosis, consequently predicting Sub-CVD (28, 34–37).

**Figure 1:**
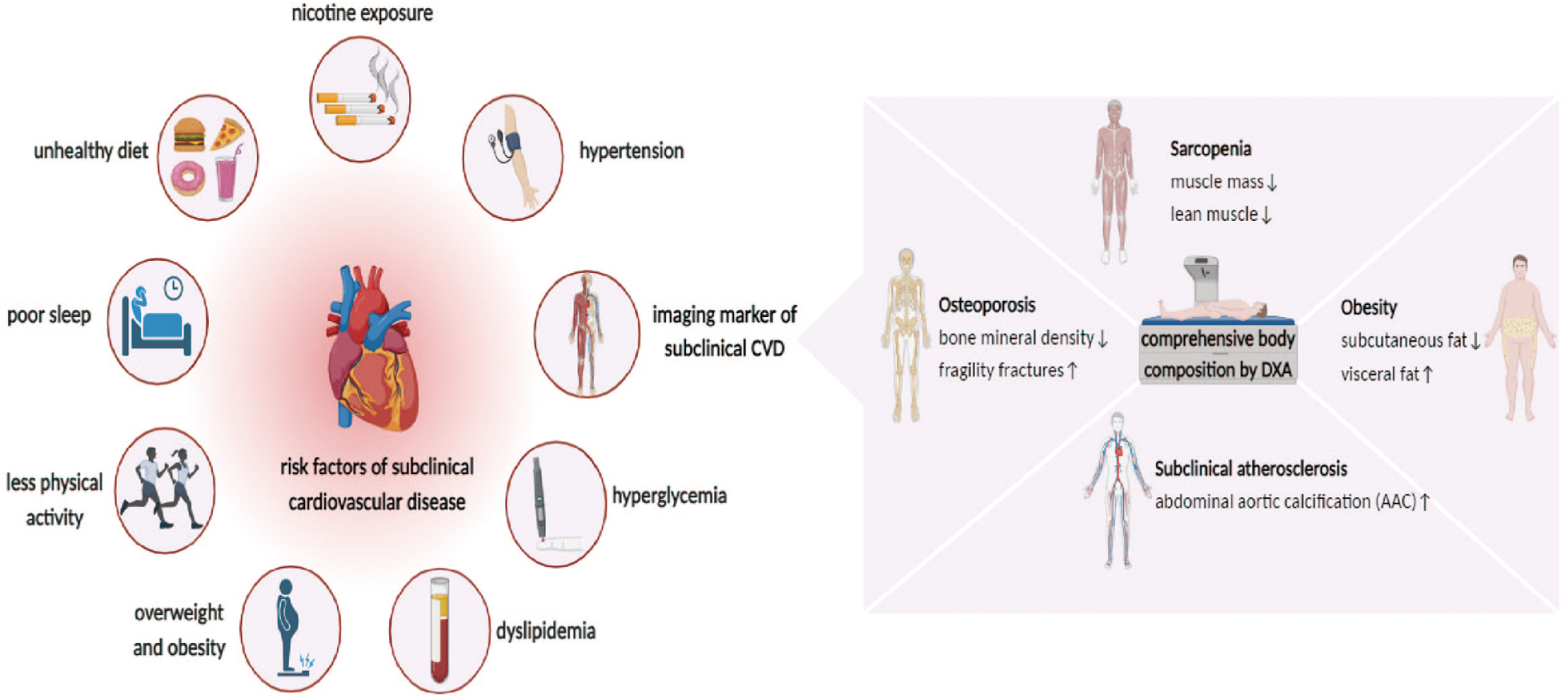
Risk factor for subclinical cardiovascular disease (Sub-CVD) and the prediction of dual-energy x-ray absorptiometry (DXA). The risk factor of Sub-CVD include lifestyle indicators such as unhealthy diet, nicotine exposure, poor sleep, and reduced physical activity, and clinical indicators such as hypertension, hyperglycemia, dyslipidemia, overweight, and obesity. It further highlights the utility of DXA in providing a comprehensive assessment of abdominal aortic calcification (AAC) and body composition, such as osteoporosis, sarcopenia, and obesity, which is instrumental in the early prediction and management of Sub-CVD, emphasizing its expanded role in cardiovascular risk assessment beyond bone health.

Typically, physical examinations, blood tests, and imaging modalities are used to diagnose Sub-CVD (10). The thorough evaluation of body fat, sarcopenia, osteoporosis, and AAC by DXA renders it a crucial tool for predicting and assessing Sub-CVD (37–40). DXA aids in the early detection and management of cardiovascular risk by providing comprehensive data on various risk factors, potentially preventing progression from subclinical to overt CVD. The multifaceted applications of DXA are expanding beyond bone health to include a wider spectrum of cardiovascular risks (Figure 1).

## 2 Risks of subclinical CVD detected by DXA

### 2.1 Body fat

Numerous studies have emphasized the significance of adipose tissue in maintaining cardiovascular health. Systemic inflammation, atherogenic dyslipidemia, and insulin resistance are induced by excessive body fat, particularly visceral adiposity. These well-established risk factors for CVD demonstrate a substantial association between obesity and increased risk of Sub-CVD (13, 14, 27, 41). Moreover, DXA can distinguish excess visceral fat, which is a significant risk factor for Sub-CVD (27, 28, 31). Aleksandra Radecka et al. demonstrated the potential of DXA in assessing body composition, such as in Cushing's syndrome, which is directly relevant to cardiovascular risk (32).

Obesity is characterized by excessive free fatty acids and triglycerides, leading to ectopic lipid deposition in various tissues, such as the liver, skeletal muscle, and myocardium. This deposition results in insulin resistance, hypertension, metabolic syndrome, type 2 diabetes mellitus, atherosclerosis, and CVD. Obesity-related dysfunctional visceral white adipose tissue generates oxidative stress and pro-inflammatory adipokines and activates the renin-angiotensin-aldosterone system, thereby increasing cardiovascular risk. Inflammation may be a potential molecular mechanism, and systemic inflammation is induced by excessive body fat, particularly visceral fat, by secreting pro-inflammatory cytokines, tumor necrosis factor-α (TNF-α), and interleukin-6 (IL-6) from adipose tissue. These cytokines significantly influence atherosclerosis and endothelial dysfunction, which are critical components of Sub-CVDs (14, 41, 42).

Obesity is associated with insulin resistance, which serves as a precursor to type 2 diabetes mellitus and metabolic syndrome, both of which are recognized risk factors for CVD. Increased cardiovascular risk is further exacerbated by dyslipidemia and hypertension resulting from insulin resistance. Adipose tissue actively secretes adipokines such as leptin, resistin, and adiponectin. Leptin and resistin upregulate oxidative stress and pro-inflammatory cytokine production, thereby promoting atherosclerosis and inflammation. Conversely, adiponectin has anti-inflammatory properties; however, its levels decrease with obesity. Lipotoxicity, caused by ectopic fat accumulation in non-adipose tissues, leads to endothelial dysfunction and cellular damage, significantly contributing to atherosclerosis, a precursor of subclinical CVD (43). Furthermore, obesity and type 2 diabetes mellitus orchestrate changes in substrate usage, tissue metabolism, oxidative stress, and inflammation, which collectively promote myocardial fibrosis and cardiac dysfunction (15).

The whole-body scan is performed using DXA. Discovery QDR software (version 13.5.3.2) then differentiates between fat and lean tissues to generate a comprehensive body fat distribution. DXA measures total body fat with a particular focus on visceral fat, which is closely related to cardiovascular risk.

### 2.2 Sarcopenia

Sarcopenia is characterized by reduced muscle mass and dysfunction, leading to decreased physical activity. Reduction in physical inactivity can potentially promote the progression of atherosclerosis and insulin resistance. Ke Gao et al. demonstrated a correlation between muscle mass loss and the rising prevalence of CVD (17). Recent research has uncovered the molecular mechanisms underlying the relationship between sarcopenia and Sub-CVD, providing evidence for this connection (16). Sarcopenia involves a reduction in muscle mass that inhibits the synthesis of nitric oxide (NO), an essential vasodilator. Decreased NO levels contribute to vascular stiffness and hypertension (44). Furthermore, muscle loss impairs glucose and lipid metabolism, exacerbating insulin resistance, a known risk factor for CVD (43, 45–47). Elevated levels of pro-inflammatory cytokines, such as C-reactive protein, IL-1, IL-6, and TNF-α, in sarcopenia lead to mitochondrial dysfunction in skeletal muscle. This dysfunction results in increased production of reactive oxygen species, which trigger the ubiquitin-proteasome cascade and amplify muscle proteolysis (48–50). Similarly, IL-6 inhibits muscle protein synthesis and activation of the Akt/mTOR pathway by inducing insulin resistance (51–54). Sarcopenia profoundly affects cardiovascular health, accelerates CVD progression, and increases the risk of mortality.

DXA determines the muscle mass, particularly in the extremities (arms and legs), which is crucial for diagnosing sarcopenia. The evaluation involves analyzing the distribution of lean muscle mass, with sarcopenia diagnosed using specific cut-off values based on established guidelines. The ability of DXA to detect sarcopenia is significantly linked to the prediction of Sub-CVD risk (30, 38).

### 2.3 Osteoporosis

Cohort research from the UK biobank has suggested a possible correlation between DXA-determined bone health and cardiovascular risk (55). Increased cardiovascular risk is associated with osteoporosis and decreased BMD, as reported in epidemiological studies (56). Notable correlations have also been identified between fragility fractures, which often result in osteoporosis, and later onset of cardiovascular conditions, including, but not limited to, heart failure, stroke, and ischemic heart disease. Osteoporosis and Sub-CVD share common risk factors, such as aging, diabetes mellitus, obesity, smoking, sedentary lifestyle, and advancing inflammation and hormonal changes. Additionally, the accumulation of calcium in the arterial walls highlights osteoporosis as a potential indicator of Sub-CVD (55, 57–59). This relationship could fundamentally alter our understanding of osteoporosis and cardiovascular health.

Multiple mechanisms link osteoporosis to CVD, and systemic inflammation plays a key role in both conditions. The Wnt signaling pathway is critical for the maintenance of cardiovascular homeostasis. Romosozumab, which inhibits sclerostin and is used in the treatment of osteoporosis, activates the Wnt pathway and may affect cardiovascular remodeling, potentially increasing the risk of cardiovascular events. The calcium paradox describes the inverse correlation between vascular calcification and bone density. Calcium from bone deterioration can exacerbate arterial calcification, which is a risk factor for Sub-CVD. The RANK/RANKL/OPG pathway, essential for bone remodeling, is also involved in vascular calcification (60, 61). Osteoprotegerin (OPG), a RANKL decoy receptor, inhibits bone resorption and contributes to vascular disease (62–65). Additionally, vitamin D deficiency and secondary hyperparathyroidism in osteoporosis may lead to vascular calcification and endothelial dysfunction (60, 66).

Furthermore, anti-osteoporotic medications may influence CVD. Bisphosphonates, which inhibit bone resorption, may impact lipid profiles and atherogenesis. Some anti-osteoporosis drugs could have beneficial cardiovascular effects, whereas certain vasoactive agents might positively affect bone health. The interplay between these domains requires further research (12, 18).

DXA is considered the gold standard for measuring BMD and is crucial for osteoporosis diagnosis. It typically scans the spine and hip and areas where fractures are the most common. The *T-* or *Z*-score, calculated by comparing BMD values with age- and sex-adjusted normative data, helps to identify osteoporosis or osteopenia. Early osteoporosis screening with DXA may also detect Sub-CVD in its early stages (55, 56).

### 2.4 AAC

Calcium deposition on the walls of the abdominal aorta is a defining feature of AAC. This process is driven by the osteogenic transformation of vascular smooth muscle cells (VSMCs) and is influenced by various factors such as inflammation, oxidative stress, and dysregulated mineral metabolism. Calcification in the aorta can reduce elasticity, cause endothelial dysfunction, impair blood flow, and increase the risk of cardiovascular events (19, 34, 35, 67). Inflammation can stimulate VSMCs within the arterial wall to undergo osteogenic differentiation, leading to calcification. Matrix vesicles, which are extracellular vesicles derived from VSMCs, play a crucial role in initiating and propagating calcification in the arterial wall (37, 68, 69).

The molecular mechanisms underlying the role of body fat, sarcopenia, osteoporosis, and AAC in the development of Sub-CVD are intricate and multifaceted. These conditions are involved in various biochemical and cellular pathways, including inflammation, metabolic dysregulation, endothelial dysfunction, and osteogenic transformation. This complexity underscores the impact of these factors on cardiovascular health. The referenced studies provide a comprehensive understanding of these mechanisms, highlighting the importance of addressing these risk factors in the prevention and treatment of Sub-CVD.

DXA can detect AAC, a marker of Sub-CVD (34, 67, 70, 71). AAC was scored on a 0–24 point scale based on baseline lateral lumbar spine x-rays. This calcification is indicative of advanced atherosclerosis and is associated with an increased risk of cardiovascular events (34, 70).

## 3 Comparison of DXA with other non-invasive imaging tools in predicting sub-CVD

Advances in digital imaging and biomarker technologies have greatly enhanced our ability to phenotype populations, providing detailed insights into the structural and functional dynamics of the cardiac and vascular systems. Developing modern non-invasive and invasive imaging technologies has led to significant advancements in diagnostic imaging (72, 73). This progress is crucial for early disease phenotyping, risk stratification, and management of CVD. Techniques such as carotid ultrasound, coronary cardiac computed tomography (CT), magnetic resonance imaging (MRI), and positron emission tomography (PET) are essential for detecting Sub-CVD risk factors, including early atherosclerosis and myocardial perfusion anomalies (74).

Carotid ultrasound measures intima-media thickness (IMT), a crucial early biomarker of atherosclerosis and arterial fibrosis that helps predict future CVD risk (75–78). The correlation between IMT and atherosclerotic histology makes it a reliable marker. Echocardiography, another ultrasound-based technique, is crucial for detecting and monitoring cardiac remodeling. Additionally, ultrasound also assesses body composition by measuring subcutaneous fat thickness and muscle echogenicity, which indicate skeletal muscle quality (77, 79–81). Although ultrasound may not be as comprehensive as MRI or CT for these evaluations, its non-invasive nature, cost-effectiveness, efficiency, and lack of radiation make it a valuable tool in clinical settings.

CT plays a crucial role in predicting CVD and evaluating body composition (82). The coronary artery calcium (CAC) score, obtained through CT, is an essential prognostic indicator of CVD (83). This score quantifies calcium deposition in coronary arteries and serves as a marker of arterial rigidity and potential atherosclerosis. CAC scoring is vital for CVD risk stratification (84, 85). CT angiography enhances diagnostic precision by visualizing the coronary architecture, detecting severe stenosis, and identifying subclinical coronary artery disease. In body composition studies, CT is instrumental in determining total body adipose tissue and muscle mass (86, 87). However, the use of CT is often limited due to concerns regarding radiation exposure.

MRI is invaluable for forecasting CVD and analyzing body composition (88–90). High-resolution MRI effectively assesses plaque volume and composition in the carotid arteries and abdominal aorta of patients with CVD. MRI discerns plaque components, such as the fibrous cap, necrotic core, and areas of hemorrhage or calcification. MRI excels in three-dimensional (3D) assessments of adipose tissue, muscle volume, and fat fraction across various organs when analyzing the body composition. MRI exhibits high spatial resolution and reproducibility, making it the clinical gold standard for evaluating cardiac structure and function (91, 92). The ability of MRI to identify cardiac fibrosis enhances its diagnostic capability. However, concerns about cost and accessibility have limited their widespread use.

PET is a sophisticated tool in cardiovascular diagnostics, particularly for detecting microvascular dysfunction (MVD) in patients with angina without obstructive coronary disease (93, 94). PET assesses coronary flow and myocardial perfusion reserve and provides information on epicardial stenosis and MVD. It accurately quantifies myocardial blood flow, making it invaluable for diagnosing MVD and evaluating cardiac perfusion in various cardiac pathologies. Although PET offers improved diagnostic accuracy for MVD through a comprehensive analysis of myocardial perfusion, limitations such as reduced temporal resolution and radiation exposure must be considered (95, 96).

DXA is a multifunctional imaging modality that offers a comprehensive assessment of body composition while minimizing radiation exposure. It is crucial for evaluating early-stage CVD risk factors, including osteoporosis, sarcopenia, and obesity (26, 28, 29, 31). DXA can also detect AAC, which is a precursor to coronary artery calcification and is significantly associated with CVD mortality, incident coronary heart disease, myocardial infarction, and stroke (34, 37, 97). Electron beam and multidetector CT are exceptionally precise for quantitative assessment, and the Agatston score is used to estimate the AAC. AAC is a readily available imaging marker that can be used to diagnose early stages of CVD. AAC can also be identified using MRI and transesophageal echocardiography. However, x-ray-based imaging technologies, including DXA, are most commonly used for detecting and diagnosing AAC owing to their accessibility and relatively low radiation exposure.

In conclusion, each imaging modality offers distinct advantages and disadvantages for predicting Sub-CVD. DXA has been recognized for its comprehensive assessment of body composition and minimal radiation exposure. However, CT and MRI provide a more direct visualization of cardiac structures and atherosclerosis, which can be crucial for detailed diagnostics. Ultrasound presents a balance between accessibility, safety, and functional assessment but does not offer the same level of comprehensive risk profiling as DXA (Table 1). The choice of imaging tool should be based on the specific clinical scenario, available resources, and the patient's overall risk profile.

**Table 1:** Comparison of non-invasive imaging tools for predicting Sub-CVD.

Imaging technology	CVD Risk factors assessed	Key advantages	Limitations
UT	Intima-media thickness Arterial fibrosis Cardiac remodeling Body composition	Non-invasive Affordable Safe	Less detailed High technical requirements for operators
CT	Coronary artery calcium score Visualizes coronary anatomy Body composition	High detail in body composition	Radiation exposure
MRI	Plaque volume Myocardial fibrosis 3D measurements of adipose tissue Muscle mass Cardiac structure/function Myocardial fibrosis	High spatial resolution Detailed cardiac function	High cost Limited accessibility
PET	Microvascular dysfunction Coronary flow reserve Myocardial perfusion reserve	High accuracy in blood flow measurement Diagnostic power	Reduced temporal resolution Radiation exposure
DXA	Osteoporosis Sarcopenia Obesity Abdominal aortic calcification	Comprehensive body composition analysis Low radiation	Limited indirect cardiac structure visualization

UT, ultrasound; CT, computed tomography; MRI, magnetic resonance imaging; PET, positron emission tomography; DXA, dual-energy x-ray absorptiometry.

## 4 Future perspectives

With advances in artificial intelligence and modern medicine, many researchers have investigated the critical function of machine learning (ML) in predicting Sub-CVD. Numerous studies have suggested that ML, particularly deep learning (DL), could improve the predictive and evaluative skills of DXA for Sub-CVD (98–101). ML models can integrate diverse datasets, including demographic, lifestyle, and disease history data, with detailed DXA data to more accurately forecast the progression of Sub-CVD. The predictive potential is critical for the timely management of Sub-CVD. By combining ML with DXA, personalized risk profiles for cardiovascular diseases can be developed. Algorithms that analyze individual DXA scans along with broader clinical data can facilitate tailored risk stratification, thereby enhancing the precision of preventive measures.

Conventional risk prediction scores are frequently employed in cardiovascular risk assessment and are supported by several national cardiovascular authorities, as well as by the World Health Organization. Commonly used scores for predicting CVD, such as the Framingham risk score, QRISK, systematic coronary risk evaluation, UK prospective diabetes study risk engine, and cardiovascular health score (2, 102–104), are combined with traditional risk factors, such as age, sex, ethnicity, socioeconomic status, family history, diet, physical activity, nicotine exposure, sleep health, body mass index, lipid profile, blood glucose, and blood pressure. Traditional scores, which are derived from statistical models based on cohort studies, employ set algorithms that do not account for the expansion of patient data. Conversely, ML operates without predetermined rules, utilizes data dynamically, and is independent of statistical assumptions, thereby enabling the discovery of complex data interactions. ML, encompassing supervised, unsupervised, and reinforcement learning, differs from traditional methods in that it focuses on predictive accuracy over hypothesis-driven inference.

ML offers diverse and powerful techniques for predicting CVDs. Supervised learning algorithms, such as neural networks and decision trees, efficiently exploit annotated data to forecast CVD by leveraging recognized risk variables. Unsupervised learning is paramount in identifying novel risk factors by unveiling hidden patterns within the data. Reinforcement learning is promising for the development of individualized treatment techniques via iterative trial-and-error methodologies (101, 105). DL, a subset of ML, excels in analyzing medical images and detecting complex indicators of Sub-CVD (106, 107). ML technology can predict and manage cardiovascular health, which is a significant advancement.

**Figure 2:**
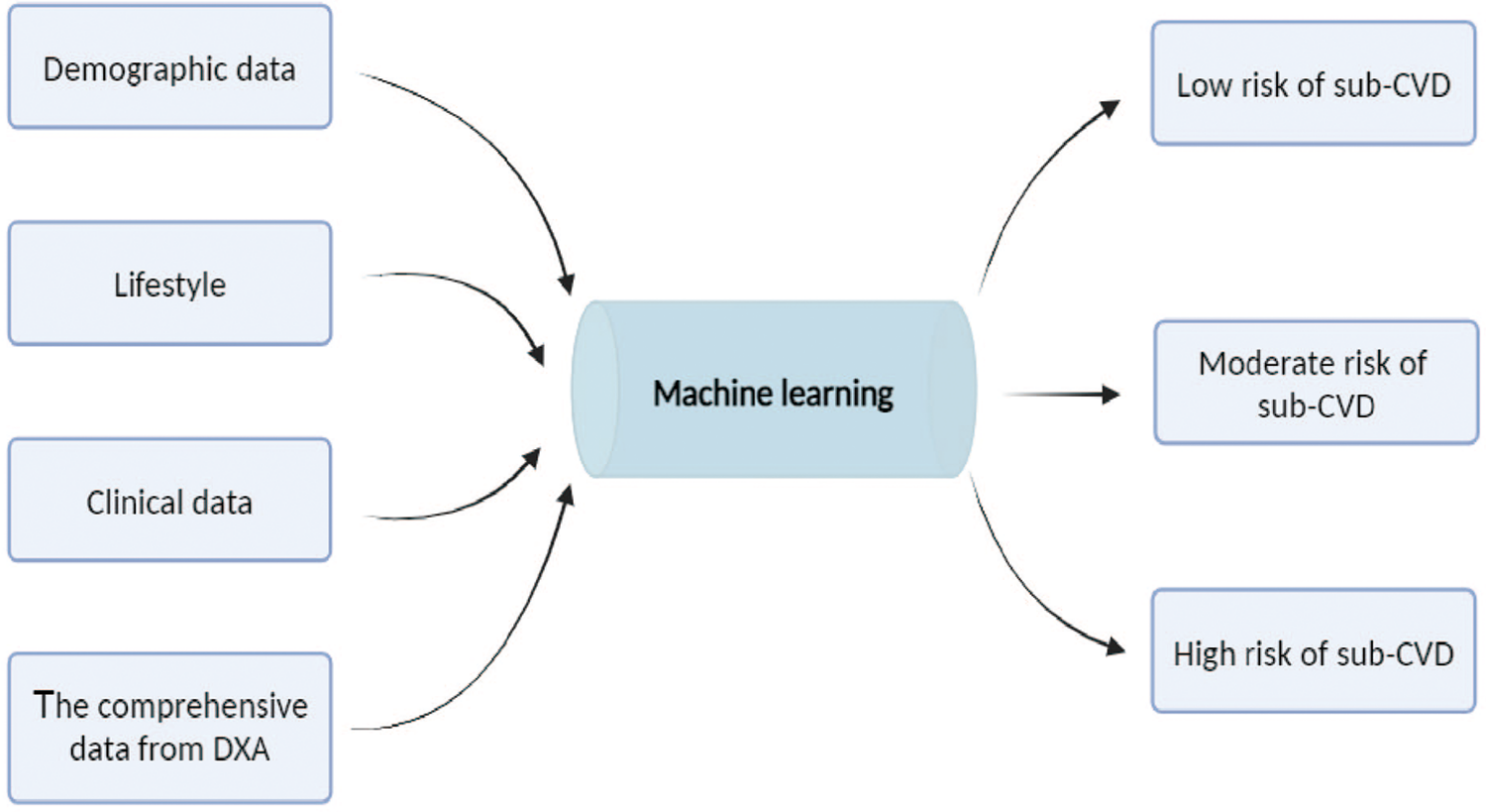
Machine learning model for cardiovascular risk stratification. Combining demographic data (such as age, gender, race, education, marital status and financial status), lifestyle (such as height, weight, diet, alcohol consumption, nicotine exposure, sleep, and physical activity and clinical indicators, hypertension, hyperglycemia, dyslipidemia, overweight, and obesity), clinical data (such as abdominal circumference, hip circumference, body mass index, blood pressure, glycemia, and lipidemia) and the comprehensive data from dual-energy x-ray absorptiometry (DXA) to the machine learning model. The model process these inputs to categorize individuals into three risk groups for subclinical cardiovascular disease (Sub-CVD): low, moderate, and high risk. This predictive model aims to enhance early detection and intervention strategies for Sub-CVD.

The integration of ML, particularly DL, with DXA can enhance diagnostic precision and personalize risk assessment, thereby making a substantial contribution to proactive and individualized patient care in cardiology (Figure 2).

## 5 Conclusion

Our review highlights the distinct capacity of DXA to precisely assess body composition, particularly body fat and lean muscle mass, which are key indicators of cardiovascular health. Besides, its effectiveness in detecting osteoporosis and AAC establishes it as a vital instrument for the stratification and management of Sub-CVD.

In summary, DXA has emerged as a diagnostic tool essential in predicting cardiovascular health (11, 38, 74). This review also underscores the necessity of a multidisciplinary approach for managing Sub-CVD, advocating the use of DXA alongside other diagnostic tools and clinical evaluations. Such an integrative strategy ensures a more comprehensive cardiovascular risk assessment, fostering more effective prevention and management tactics.
